# Enzymes as Additives or Processing Aids in Food Biotechnology

**DOI:** 10.4061/2010/436859

**Published:** 2011-04-19

**Authors:** Raffaele Porta, Ashok Pandey, Cristina M. Rosell

**Affiliations:** ^1^Department of Food Science, University of Naples Federico II, Portici, 80055 Napoli, Italy; ^2^Biotechnology Division, National Institute for Interdisciplinary Science and Technology, CSIR, Trivandrum, Kerala 695019, India; ^3^Institute of Agrochemistry and Food Technology (IATA-CSIC), 46980 Paterna, Valencia, Spain

Essential in the metabolism of all living organisms, the enzymes are increasingly used to drive chemical reactions outside their natural localization. In particular, the use of the biocatalysts as food additives and in processing raw materials has been practiced for a long time. In fact, enzymatic preparations from the extracts of plants or animal tissues were used well before much was known about the nature and properties of enzymes. 

Food industry is constantly seeking advanced technologies to meet the demand of the consumers, and enzymes have long been used by the industrial product makers as major tools to transform the raw materials into end-products. Their clean label (GRAS, generally recognized as safe) consideration from the legal point of view has prompted their extensive use in food technology. When purified and added to food preparations, several enzymes are able to improve their flavor, texture, digestibility, and nutritional value. However, it was not until the mid of the past century that the rapid development in protein technology occurred, and only in the last 30 years, the use of commercial enzymes has grown in the food industry, progressively becoming an important aspect of the manufacturing of meat, vegetables, fruit, baked goods, milk products, and both alcoholic and nonalcoholic beverages. As a matter of fact, an increasing number of articles, mostly describing the enhanced product yields, have been published during the last ten years, both in food and beverage manufacturing. Moreover, since it is desirable in different branches of food technology to change the physical and chemical properties of protein, many previously unexplored enzymes are currently employed to produce a variety of foods in which the biocatalysts replace potentially carcinogenic or otherwise harmful chemicals. This includes also new methods in which the characteristics of natural products are altered to fit the nutritional or technological needs changing. 

The economic benefit of using technical enzyme preparations lies in lowered process costs, in the reduction of the environmental impact by making use of renewable resources, and often in increasing the quality of the products. Also, preservation makes a significant impact on the quality of food as well of beverages. It is well known, for example, that modern processes convert juices into concentrates that, except for aroma, can be stored for a long time without loss in quality. Stabilizing flavor and color is also an example of improved preservation. Finally, the advent of biotechnology has also allowed significant refinements in the methodologies offering unpredictable solutions to many persistent problems and opening up exciting new possibilities. Among these, enzymes are proposed as exemplary agents of “green” technology since they can also be used either to treat the biological wastes or to prevent their formation. Currently used enzymes sometimes originate in animals and plants but most come from a range of beneficial microorganisms. Thus, numerous purified enzymes are now being widely used not only in food processing but also as food additives. In this respect, it is noteworthy that the enzymes, like all proteins, can cause reactions only when people have been sensitized through exposure to large quantities. Therefore, since their levels in the food are generally very low, the enzymes are highly unlikely to cause allergies. 

This special issue of Enzyme Research is devoted to contribute to highlight some expanding fields of enzyme applications in food technology, mostly explaining how some different biocatalysts bring advantages in some food processing improvement and innovation. It comprises six review articles and three research articles. The first review article is a condensed and concise overview on the applications of enzymes in food and feed processing, outlining the development of better biocatalysts through microbial screening, protein engineering, and immobilization techniques. The second review article summarizes the nutritional, technological, and environmental advances in meat products and, in particular, the application of the proteolytic enzymes, phytases, and transglutaminase in the meat industry. Transglutaminase, as well as bacterial-derived endopeptidases, are the subject of the third review article which reports the most recent developments of the attempts to detoxify gluten. The fourth and the fifth review articles describe, respectively, the uses of laccases as additives in food and beverage processing and the production, function, and applications in food industry of fungal laccases. The last review article has been focused on the different types of techniques used for the immobilization of *β*-galactosidase and its potential applications in food and dairy processing industries. The three research articles describe (i) a new glucose biosensor based on a screen-printed carbon electrode modified by glucose dehydrogenase immobilized on its surface, (ii) the preparation of antioxidant hydrolysates of honeybee-collected pollen by using proteinase and aminopeptidases of plant origin, and (iii) the characterization of a potential food-grade leucine aminopeptidase extracted from kiwifruit.

We sincerely hope that the present volume may represent only the first of a special issue series in which Enzyme Research will periodically stimulate authors to publish the highlights and original research articles reporting how enzymes bring new advantages in food preparation improvement and innovation.

*Raffaele Porta* *Raffaele Porta* *Ashok Pandey* *Ashok Pandey* *Cristina M. Rosell* *Cristina M. Rosell*

